# Increased proliferation and differentiation capacity of placenta-derived mesenchymal stem cells from women of median maternal age correlates with telomere shortening

**DOI:** 10.18632/aging.203724

**Published:** 2021-11-29

**Authors:** Erika N. Guerrero, Shantal Vega, Cindy Fu, Ruth De León, Davis Beltran, Mairim Alexandra Solis

**Affiliations:** 1 Stem Cell Research Group, Department of Research in Sexual and Reproductive Health, Gorgas Memorial Institute for Health Studies, Panama City, Republic of Panama; 2 Universidad de Panamá, Panama City, Republic of Panama; 3 Universidad Latina de Panamá, Panama City, Republic of Panama; 4 Department of Research in Virology and Biotechnology, Gorgas Memorial Institute for Health Studies, Panama City, Republic of Panama; 5 Sistema Nacional de Investigación, SENACYT, Panama City, Republic of Panama

**Keywords:** mesenchymal stem cells, proliferation, differentiation, multipotent markers, telomere length

## Abstract

Mesenchymal stem cells (MSCs) experience functional decline with systemic aging, resulting in reduced proliferation, increased senescence, and lower differentiation potential. The placenta represents a valuable source of MSCs, but the possible effect of donor age on the properties of placenta-derived mesenchymal stem cells (PDMSCs) has not been thoroughly studied. Thus, the aim of this study was to underscore the effect of maternal age on the biological characteristics and stemness properties of PDMSCs. PDMSCs were isolated from 5 donor age groups (A: 18-21, B: 22-25, C: 26-30, D:31-35 and E: ≥36 years) for comparison of morphological, proliferative and differentiation properties. The pluripotency markers NANOG, OCT4, and SSEA4, as well as multipotency and differentiation markers, showed higher expression in PDMSCs from mothers aged 22-35 years, with up to a 7-fold increase in adipogenesis. Cumulative population doubling, cell growth curves, and colony-forming unit-fibroblast assays revealed higher self-renewal ability in donors 26-30 years old. An increase in the proliferative characteristics of PDMSCs correlated with increased telomere shortening, suggesting that shorter telomere lengths could be related to cellular division rather than aging. A clear understanding of the effect of maternal age on MSC regenerative potential will assist in increasing the effectiveness of future cell therapies.

## INTRODUCTION

Since their discovery, mesenchymal stem cells (MSCs) have become one of the most promising types of stem cells currently approved for use in clinical applications [1–3]. Among them, placenta-derived mesenchymal stem cells (PDMSCs), named for their fetal niche, have proven to possess superior plasticity in several clinical trials, including those related to cardiovascular diseases, pulmonary fibrosis, ischemic stroke, and type 2 diabetes, among others [4–6]. However, donor age has been demonstrated to affect the regenerative capacity of MSCs from different sources [7], such as those derived from dental pulp, bone marrow, and the placenta [8–10]. At young ages, the capacity of MSCs for proliferation and differentiation, as well as the expression of lineage surface markers, is greater than that of MSCs from older individuals [11, 12]. Compared to BM-MSCs from older donors, bone marrow-derived MSCs from young donors have been reported to exhibit higher proliferative capacity but no differences in immunological markers [13, 14]. A lower yield, decreased differentiation capacity, and an increased occurrence of senescence have been observed for MSCs from older donors [15]. Studies of dental pulp-derived MSCs have revealed an age-dependent decrease in osteogenic differentiation, with the lowest potential observed in donors aged >60 years, followed by those aged 20-40 years and those aged 7-12 years [16]. Thus, the effect of aging on the biological properties of stem cells has been the focus of a great number of studies [17, 18]. Unfortunately, the impact of maternal age on *in vitro* cultured PDMSCs has not yet been evaluated. Thus, in this study, we aimed to understand the effect of maternal age on self-renewal capacity, proliferation, multipotent and pluripotent marker expression, and differentiation potential in five age groups, namely, groups A (18-21 years), B (22-25 years), C (26-30 years), D (31-35 years), and E (36 years or older), and to what extent these parameters correlate with telomere length.

## RESULTS

### PDMSCs isolated from donors 22-35 years of age showed higher self-renewal properties and proliferative capacity

**Figure 1:**
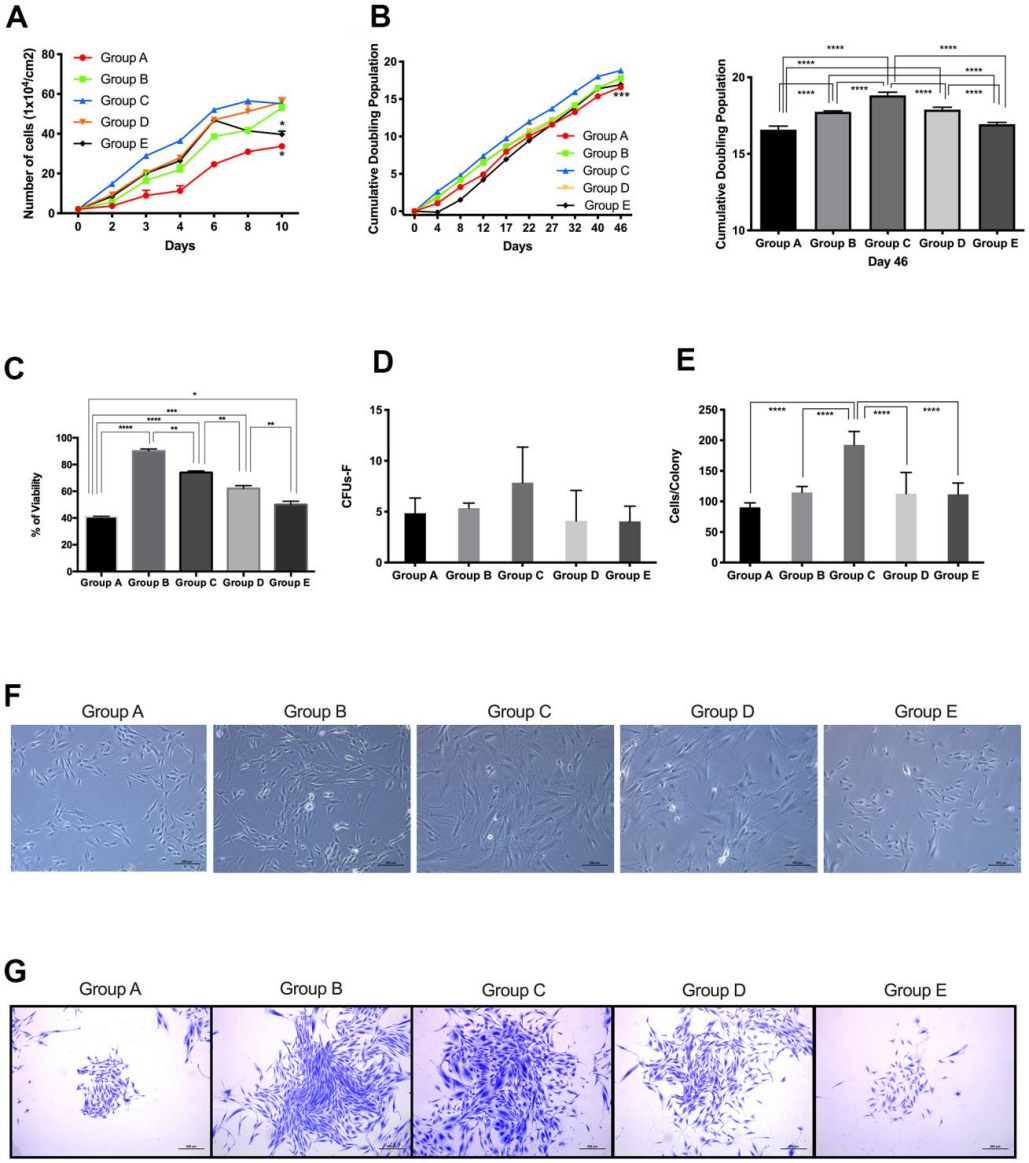
**Proliferative capacity of placenta-derived mesenchymal stem cells from different maternal age group.** (**A**) Cell growth curve showing 5 age groups. Cells were seeded at 1x10^4^ per cm^2^ in 24-wells plates and counted every 48 hours for 10 days. (**B**) Cumulative Doubling Population curve showing 5 age groups. Cells were seeded at 1x10^4^ per cm^2^ in 24-wells plates and subculture every 4 to 5 days. Population Doublings (PD) was calculated using the formula ΔPD= log(total cell number/initial cell number)log2. Each dot represents one passage. Bar graph represents the cumulative population doubling among the 5 age groups at day 46. (**C**) MTT cell proliferation assay performed using Thiazolyl Blue Tetrazolium Bromide in a 96-well ELISA plate. (**D**) Graph showing Colony Forming Unit-Fibroblast (CFU-F) assay of 5 age group. 1x10^3^ cells were seeded in 12-wells plates and allowed to grow for 8 days, before staining with Crystal Violet. Colonies with more than 40 cells were counted. (**E**) Graph showing the number of cells in colonies counted for the CFU-F of 5 age groups. (**F**) Representative image showing cellular morphology of 5 age groups at 4 days of culture. (**G**) Images represent crystal violet staining in cells from each maternal age group. Group A: 18-21; Group B: 22-25; Group C: 26-30; Group D: 31-35; Group E: 36 and over. *, *p*<0.05; **, *p*<0.01; ***, *p*<0.0005; ****, *p*<0.0001. Scale bar 200 μm.

To analyze the effect of maternal age on the self-renewal and proliferative capacity of PDMSCs, we performed CGC, CPD and CFU-F assays. The results showed that cells derived from maternal age group A presented a slower growth rate than cells from the higher age groups (Figure 1A). Groups B, C, D and E presented a similar growth pattern until day 6, when group E showed a significant (*p*<0.05) decline in growth rate compared to those of groups B, C and D.

CPD data showed that groups A and E presented significantly (*p*<0.0005) lower doubling capacity than cells from groups B, C and D (Figure 1B). Results from the MTT (3-[4,5-dimethylthiazol-2-yl]-2,5 diphenyl tetrazolium bromide) assay confirmed our observations of PDMSCs isolated from donors 22-35 years of age showing higher self-renewal properties and proliferative capacity (Figure 1C).

Following cell expansion, adherent PDMSCs obtained from all 5 maternal age groups revealed a similar spindle-shaped, fibroblastic morphology, which corresponds to the typical appearance of MSCs in primary culture at passage 4 (Figure 1F).

CFU-F assays were carried out to quantify the colony formation capacity of cells from all 5 maternal age groups. Cells were seeded at 1x10^3^ and allowed to grow for 8 days; after staining with crystal violet, colonies with cell numbers <40 were counted. The results showed that Group C presented higher colony formation capacity (Figure 1D, 1E) than the other groups, whereas groups A and E showed the lowest number of colonies formed. Furthermore, when the number of cells per colony was counted (Figure 1G), Group C consistently showed more cells per colony than the other maternal age groups. Colony formation capacity correlated with the results obtained for CGC and CPD. Taken together, these results demonstrated that PDMSCs isolated from mothers aged 22-35 years have better self-renewal and proliferative capacity than cells isolated from mothers aged 18-21 or >36 years.

### PDMSCs isolated from donors 22-35 years of age presented greater expression of pluripotency and multipotency markers

**Figure 2:**
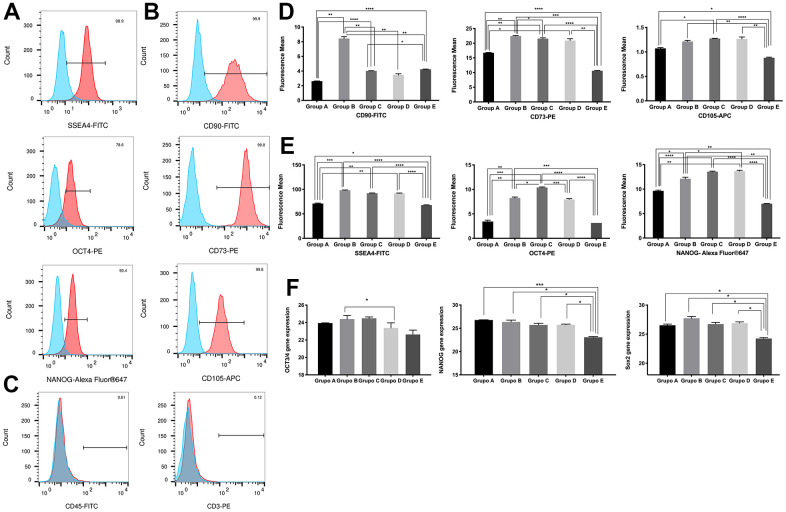
**Characterization of placenta-derived mesenchymal stem cells based on surface and nuclear markers expression from different maternal age group.** (**A**) Representative histograms of detection by fluorescence-activated cell sorting (FACS) demonstrates positive expression of three MSC pluripotency intracellular markers. Populations are more than 95% positive for SSEA4 and NANOG. (**B**) Representative histograms of detection by FACS demonstrates positive expression of three MSC multipotency surface markers. Populations are more than 99% positive for CD90, CD73 and CD105. (**C**) Representative histograms showing no detection by FACS demonstrates no expression of the immunologic markers CD45 and CD3. Populations are more than 99% negative for CD45 and CD3. (**D**) Graphs of difference in mean fluorescence of MSC pluripotency intracellular markers of all 5 age groups. (**E**) Graphs of difference in mean fluorescence of MSC multipotency surface markers of all 5 age groups. (**F**) Graphs showing difference in expression of MSC pluripotency intracellular transcripts NANOG, OCT4 and SOX2 gene expression measure by Real Time PCR of all 5 age groups. Group A: 18-21; Group B: 22-25; Group C: 26-30; Group D: 31-35; Group E: 36 and over. *, *p*<0.05; **, *p*<0.01; ***, *p*<0.0005; ****, *p*<0.0001.

Since the expression of multipotency and pluripotency markers is a major characteristic of PDMSCs, we next decided to examine the effect of maternal age on these markers. Flow cytometry results revealed specific staining for each of the positive markers of MSC stemness (Figure 2A, 2B), while the expression of the negative markers CD45 and CD3 (Figure 2C) was undetectable. No expression was detected in isotype controls (Supplementary Figure 1). We then measured the difference in the mean fluorescence expression of pluripotency and multipotency markers among all 5 age groups (Figure 2D, 2E) and observed significantly (*p*<0.0001) reduced expression in age groups 18-21 and >36 compared to age group 22-35.

To ensure that the observed changes in intracellular pluripotency marker expression were consistent at the transcriptional level, we validated our findings by RT-qPCR. Gene expression analysis of OCT4, NANOG and Sox2 showed significantly (*p*<0.05) reduced expression in the 18-21 and >36 age groups compared to the 22-35 age group, consistent with our flow cytometry observations (Figure 2F).

Overall, while all age groups of PDMSCs maintained their multipotency under standard culture conditions, cells from groups A and E presented reduced plasticity given their decreased marker expression compared to that of groups B, C, and D.

### PDMSCs isolated from donors 26-35 years of age have higher mesenchymal lineage differentiation potential

**Figure 3:**
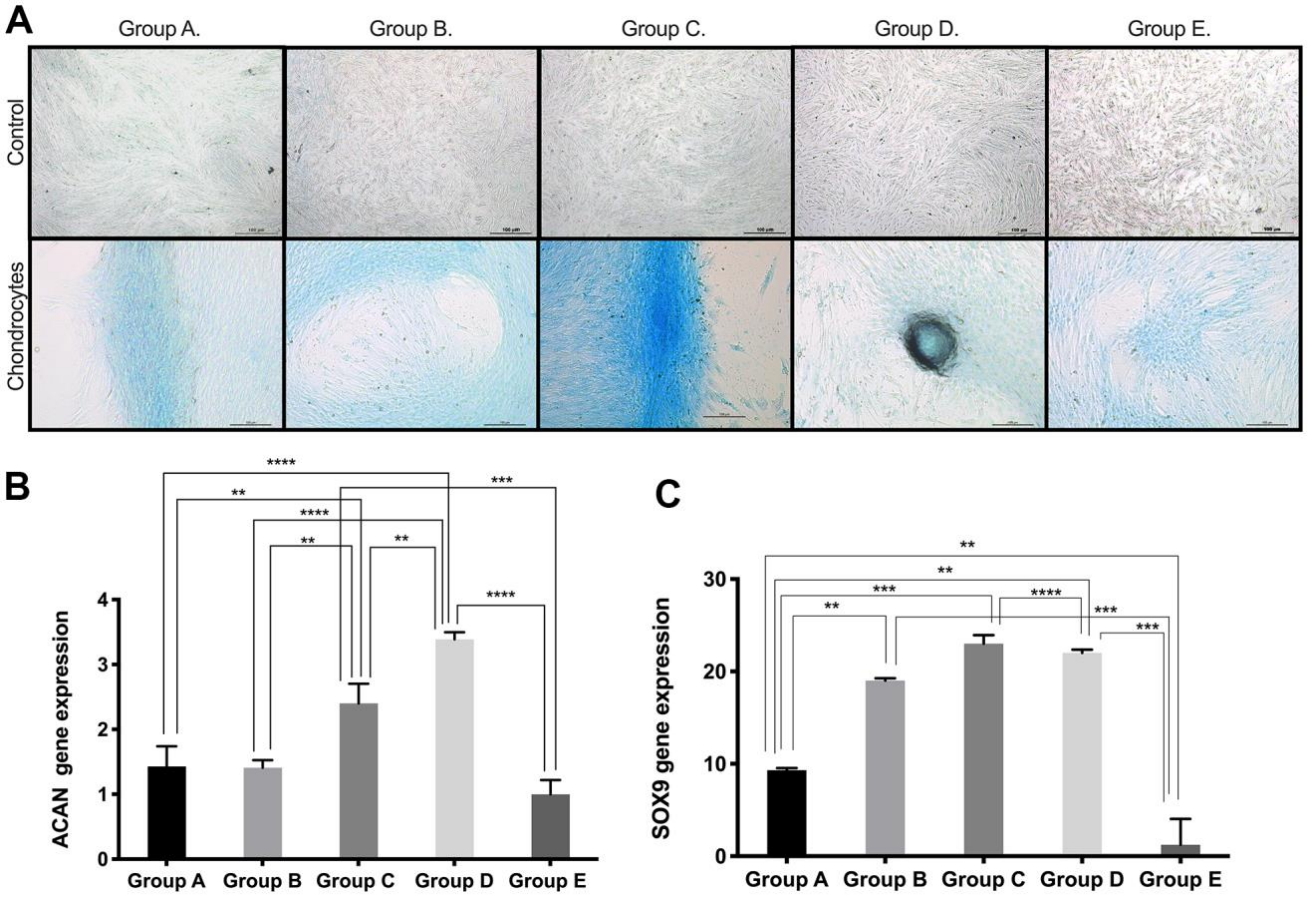
**Chondrogenic differentiation ability of PDMSCs from different maternal age group.** (**A**) Representative image of chondrogenic differentiation of all 5 age groups. Upper panel showed that undifferentiated cells did not retain Alcian blue staining. Lower panel showed that differentiated cells were positive to Alcian blue staining. Real Time PCR performed to measure chondrocyte markers gene expression of (**B**) Aggrecan (ACAN) and (**C**) SOX9 of all 5 age groups. Group A: 18-21; Group B: 22-25; Group C: 26-30; Group D: 31-35; Group E: 36 and over. *, *p*<0.05; **, *p*<0.01; ***, *p*<0.0005; ****, *p*<0.0001. Scale bar 200 μm.

**Figure 4:**
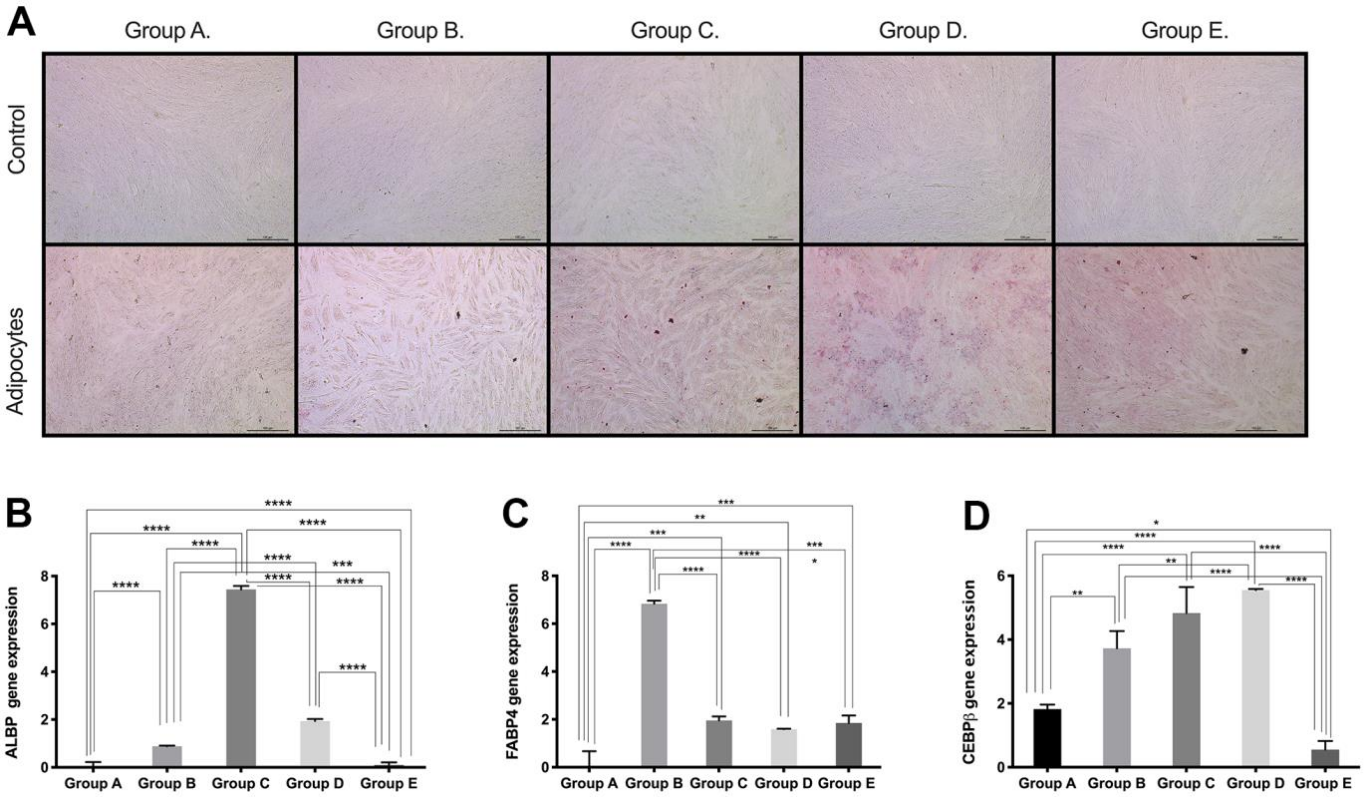
**Adipogenic differentiation ability of PDMSCs from different maternal age group.** (**A**) Representative image of adipogenic differentiation of all 5 age groups. Upper panel showed that undifferentiated cells did not retain Oil-O Red staining. Lower panel showed that differentiated cells were positive to Oil-O Red staining. Real Time PCR performed to measure the gene expression of (**B**) Adipocyte lipid binding protein (ALBP) (**C**) Fatty acid-binding protein 4 (FABP4), and (**D**) CCAAT/enhancer-binding protein beta (CEBPB) of all 5 age groups. Group A: 18-21; Group B: 22-25; Group C: 26-30; Group D: 31-35; Group E: 36 and over. *, *p*<0.05; **, *p*<0.01; ***, *p*<0.0005; ****, *p*<0.0001. Scale bar 200 μm.

**Figure 5:**
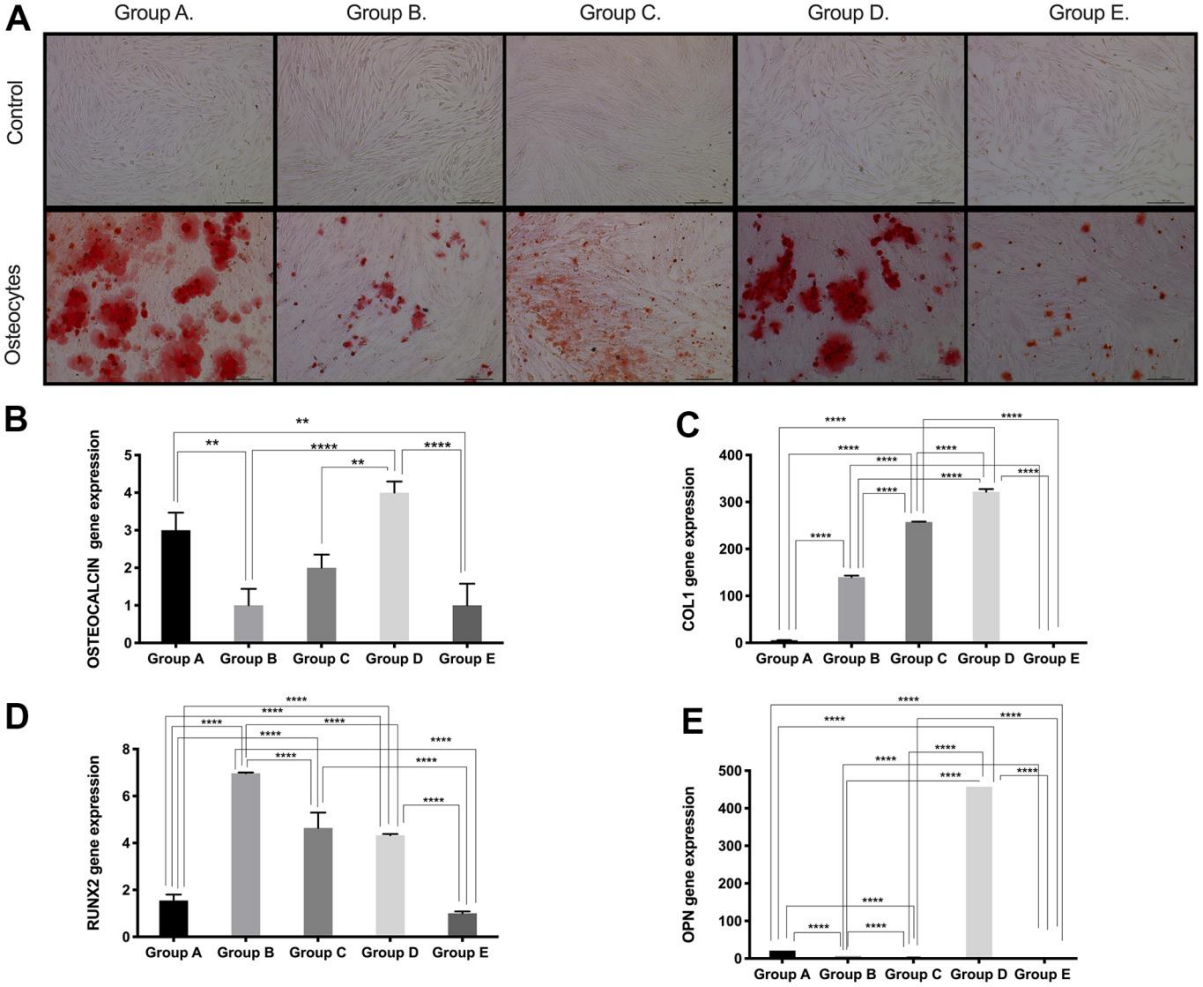
**Osteogenic differentiation ability of PDMSCs from different maternal age group.** (**A**) Representative image of Osteogenic differentiation of all 5 age groups. Upper panel showed that undifferentiated cells did not retain Alizarin staining. Lower panel showed that differentiated cells were positive to Alizarin staining. Real Time PCR performed to measure the gene expression of (**B**) Osteocalcin, (**C**) Collagen type 1 (COL1), (**D**) Runt-related transcription factor 2 (RUNX2), and (**E**) Osteopontin (OPN) of all 5 age groups. Group A: 18-21; Group B: 22-25; Group C: 26-30; Group D: 31-35; Group E: 36 and over. *, *p*<0.05; **, *p*<0.01; ***, *p*<0.0005; ****, *p*<0.0001. Scale bar 200 μm.

Based on the results obtained for the expression of pluri- and multipotency markers as an indicator of cell plasticity in PDMSCs among all 5 groups, we then induced mesenchymal multilineage differentiation to understand the effect of maternal age. All groups included in this study were differentiated into chondrogenic, adipogenic and osteogenic lineages. Chondrocytes stained positive for Alcian Blue, in contrast to undifferentiated controls (Figure 3A), indicating the synthesis of proteoglycans by chondrocytes. We then confirmed chondrogenic differentiation by RT-qPCR analysis of the gene expression of the chondrogenic markers Aggrecan (ACAN) and the transcription factor SOX9. ACAN showed significantly (*p*<0.0001) higher expression (~2.5- to ~3.5-fold change) (Figure 3B) along with a >20-fold increase in the transcription factor SOX9 expression (Figure 3C), in groups C and D compared to the other groups, consistent with the Alcian blue staining. Oil Red O, a dye that stains triglyceride and lipid deposits, showed positive staining for adipocyte-differentiated cells, in contrast to the undifferentiated controls (Figure 4A). Adipocyte lipid-binding protein (ALBP) gene expression confirmed adipogenic differentiation, with significantly (*p*<0.0.0001) higher expression (~7-fold change) in cells from group C than in cells from the other groups (Figure 4B), as well a 6-fold increase of the fatty acid-binding protein 4 (FABP4) and >3.8-fold of CCAAT/enhancer-binding protein beta (CEBPB) in group B, C, or D (Figure 4C, 4D). Osteocytes stained positive for Alizarin Red (Figure 5A), a stain that identifies calcium-containing osteocytes in differentiated culture. Osteocalcin gene expression confirmed osteocyte differentiation (Figure 5B) and showed significantly (*p*<0.0.001) higher expression in groups D (~4-fold change), A (~3-fold change), and group C (~2-fold change) than in the other groups. The expression of other osteogenic differentiation markers, such as collagen type 1 (COL1) and Runt-related transcription factor 2 (RUNX2), were highly upregulated in groups B, C, and D (Figure 5C, 5D), with more than 400-times increase of osteopontin (OPN) gene expression in group D (Figure 5E). Taken together, the results showed that although groups B, C and D presented higher expression of pluri- and multipotency markers, after induction of differentiation, groups B, C and D showed the highest differentiation potential, and group A also showed an elevated capacity for osteogenic differentiation compared to that of the undifferentiated controls.

### Telomere length was negatively correlated with PDMSC self-renewal and proliferative capacity in all 5 maternal age groups

**Figure 6:**
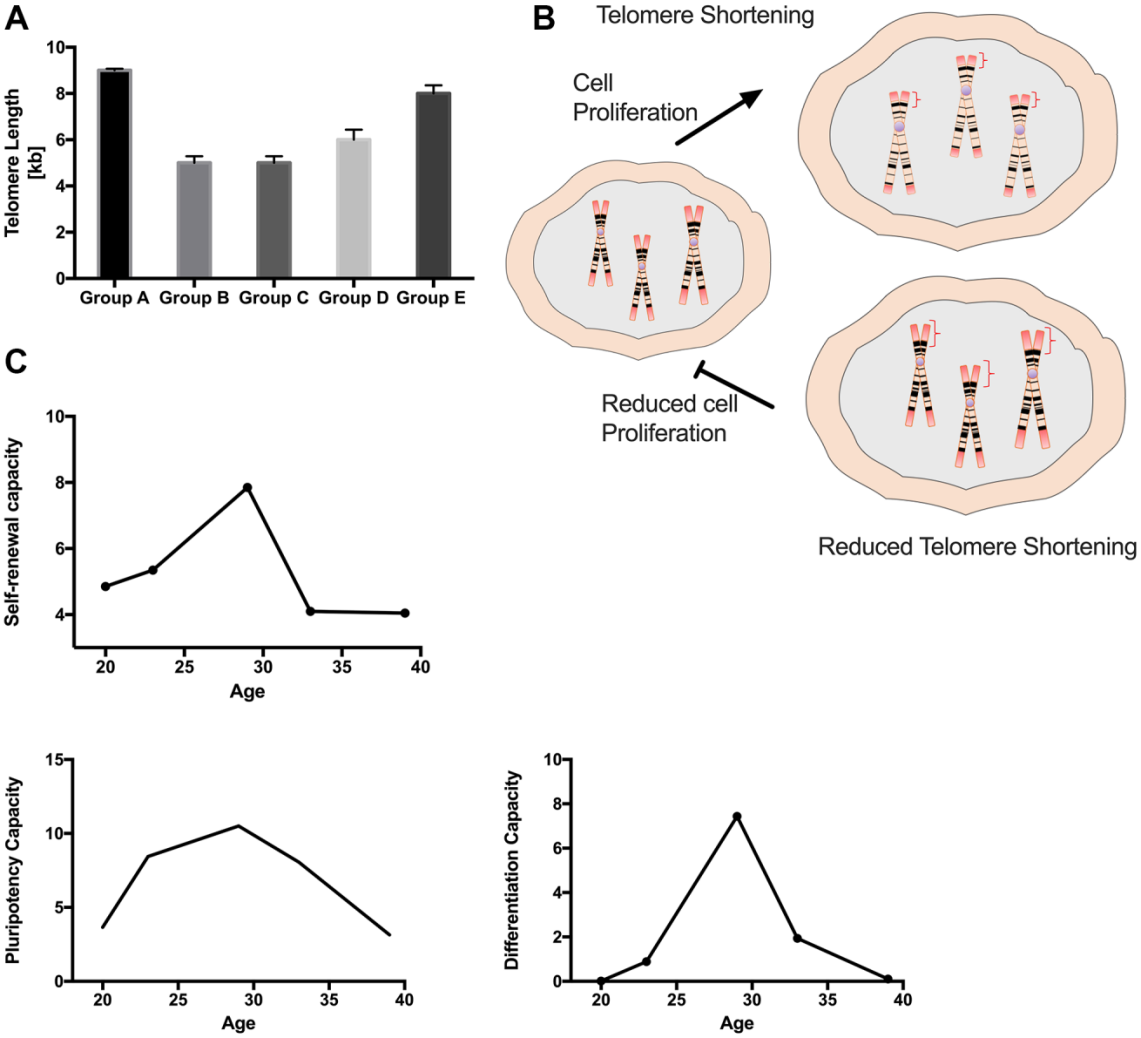
**Maternal age influences PDMSCs telomere length and correlates with MSCs characteristics.** (**A**) Graph showing the telomere length for all 5 age groups (**B**) Schematic representation of hypothesis for reduced telomere shortening. (**C**) Graph Showing correlation between age factor and cellular self-renewal capacity, cellular multipotency capacity and cellular differentiation capacity. Group A: 18-21; Group B: 22-25; Group C: 26-30; Group D: 31-35; Group E: 36 and over. *, *p*<0.05; **, *p*<0.01; ***, *p*<0.0005; ****, *p*<0.0001.

The age-related decline in the regenerative potential of MSCs has been correlated with telomerase-related telomere elongation loss of function [19]. Based on this, we decided to investigate whether there was a correlation between increased maternal age and decreasing telomere length in PDMSCs. Telomere length was assessed through RT-qPCR. Surprisingly, the results showed that groups A and E presented longer telomeres than groups B, C and D (Figure 6A). Interestingly, when correlating telomere length with self-renewal and proliferative capacity (Figure 1), groups with reduced proliferative capacity (A and E) had longer telomere length. On the other hand, PDMSCs from mothers aged 22-35 year had higher cell proliferation and shorter telomeres, probably due to their increased cell division (Figure 6B). Measurement of the expression of p21 by RT-qPCR, revealed that no cellular senescence was present among the different maternal age groups (Supplementary Figure 2). These data suggest that the reduced telomere length in PDMSCs could be due to increased cellular proliferation rather than age-related effects. Taken together, our data demonstrated correlations of self-renewal properties, multipotency capacity and differentiation ability with maternal age (Figure 6C), showing that PDMSCs from mothers aged 22-35 years have better regenerative potential for biomedical research and clinical applications.

## DISCUSSION

**Figure 7:**
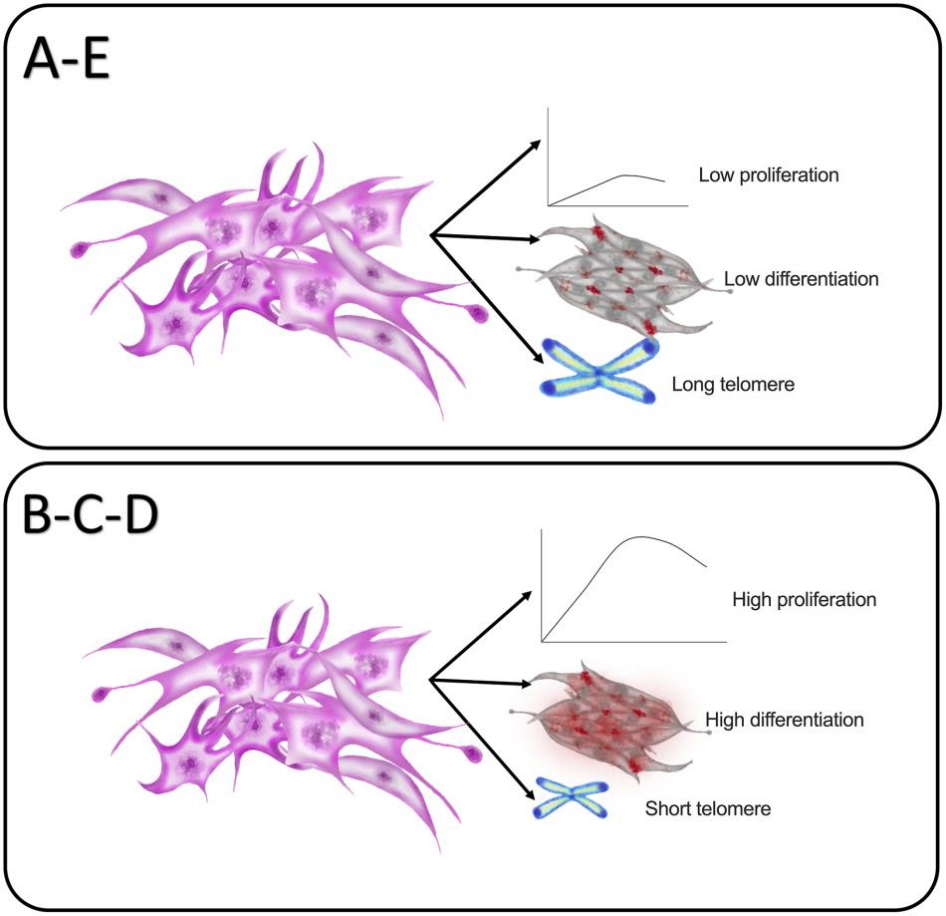
**Graphical summary of the impact of maternal age in PDMSCs cellular properties.** Group A: 18-21 and Group E: 36 and over showed reduced self-renewal and proliferative capacities with reduced lineage differentiation ability. In contrast both groups showed longer telomere length. Group B: 22-25, Group C: 26-30 and Group D: 31-35 demonstrated higher self-renewal and proliferative capacity with higher lineage differentiation ability with reduced telomere length.

MSCs are an attractive source for cell therapy owing to their unique self-renewal ability, multipotency, and ease of isolation. However, these properties may vary depending on the tissue source and donor age. We demonstrated a clear correlation between donor age and the stemness properties of PDMSCs and investigated how their biological function is affected by age (Figure 7). We evaluated PDMSC self-renewal properties and multipotency capacity in 5 donor age groups. Our results lead us to suggest that PDMSCs isolated from mothers between 22 and 35 years of age demonstrated a higher growth rate, better proliferative capacity, and successful induction of differentiation into adipocytes, osteocytes and chondrocytes. We also reported increased telomere shortening in PDMSCs isolated from maternal ages between 22 and 35 years compared to PDMSCs from younger donors (18-21 years) or older donors (36 years and older).

Advanced age has been demonstrated to be negatively correlated with cellular regenerative potential, but little is known about the correlation between the source of MSCs and donor age. Human adipose-derived MSCs from young (<30 years), adult (35-50 years) and aged (>60 years) donors have been shown to exhibit an age-related decrease in MSC frequency, cell colony formation, and cellular doubling [20], as well as a decrease in proliferation and differentiation potential [21]. Similar findings were reported in MSCs isolated from dental pulp [22], bone marrow [23] and human amniotic fluid-derived stem cells (hAFSCs) [24]. hAFSCs from donors divided into 20-29, 30-39 and 40-49 years age groups demonstrated an increase in population doubling time with age, and cells from donors of younger ages showed higher cellular proliferation [24]. In contrast to previous studies, our strategy of using smaller age ranges allowed us to underscore that PDMSCs derived from donors with median ages (22-25, 26-30, and 31-35 years) showed an increased capacity for cellular proliferation, cell doubling, and colony formation in that of cells from the younger (18-22 years) and older (>36 years) age groups, demonstrating that there is an increase in self-renewal properties at median ages, followed by a decrease in proliferation potential with further increases in age.

Several previous studies have identified stemness markers of cells isolated from placental tissue. Abumaree et al. reported CD105- and CD90-positive stem cells from chorionic villi of the human term placenta using flow cytometry [25]. Furthermore, Borzou et al. [24] demonstrated that hAFSCs from different age groups were positive for CD73, CD90 and CD105, mesenchymal stem cell markers, and negative for CD34 and CD45, hematopoietic markers, but few, if any, studies have investigated the effect of maternal age on the expression of MSC surface antigen markers and the differential expression of these markers in PDMSCs. Alrefaei et al. measured the differential expression of CD105 and CD29 via immunohistochemistry and gene expression analysis in components of umbilical cord [26] and placental [10] tissues of 5 maternal age groups; they reported a negative correlation with maternal age, with strong cytoplasmic and nuclear CD105 expression in the placental villi from 20- to 25- and 26- to 30-year-old donors and decreased expression in donors aged >31 years. These results are somewhat consistent with our results considering that the previously reported marker CD105 was measured in tissue. To our knowledge, we report for the first time how the age of maternal donors negatively affects the differential expression of the multipotency markers CD90, CD73 and CD105 by flow cytometry and the gene expression of *in vitro* cultured MSCs isolated from placental tissue. In addition, we reported differential expression of the pluripotency markers NANOG, OCT4, SSEA4, and Sox2, with PDMSCs from donors of median ages (22-35 years) presenting the highest expression. Fetal-tissue-derived MSCs present unique potency as they are able to differentiate into the three germ layers and express pluripotency markers, as widely described [27–30]. Tivanović et al. reported a comparative evaluation of pluripotency marker expression among MSCs derived from different sources, such as peripheral blood (PB-MSCs), umbilical cord (UC-MSCs), the periodontal ligament (PDL-MSCs) and adipose tissue (AT-MSCs), and observed that although MSCs from all sources expressed NANOG, OCT4, Sox2 and SSEA4, PDL-MSCs and AT-MSCs showed the highest mRNA expression of pluripotency markers [31]. However, comparative studies of differential expression among donor age groups were not reported.

Multipotency may ultimately be reflected in cellular mesodermal lineage differentiation. Regarding chondrogenic differentiation potential, we observed a significant age-related difference in groups C and D, with higher chondrogenic potential measured by Alcian Blue staining and mRNA expression of ACAN. However, studies reported in BM-MSCs showed no difference in chondrogenesis among the three age groups studied (child: 0-12 years, adult: 25-50 years and old: over 60 years) [23] possibly due to the source of MSCs [32]. Other studies have reported decreased chondrogenic differentiation potential in AT-MSCs from increasing age groups (<30, 35-55 and >60 years), which is somewhat consistent with our findings [20]. Interestingly, Choudhery et al. studied age groups (<30, 35-55 and >60 years) but did not include an analysis of samples from donors 30-35 years, which is the age group (Group D: 31-35 year) we report to show the highest chondrogenic differentiation potential (Figure 3). Our findings suggest that PDMSCs derived from donors aged 26-35 years may represent a suitable source for cell therapies for cartilage repair.

When analyzing the ability of PDMSCs to differentiate into adipocytes, we observed an increase in the expression of the adipogenesis-specific gene ALBP in groups B, C and D, with group C (26-30 years) showing a significant 7-fold increase in expression. Studies of BM-MSCs and AT-MSCs have shown a negative impact of age on adipogenic differentiation [33], whereas others have reported no difference in adipogenic differentiation with age [20]. This lack of consistent results may be due to the age ranges of the donors that were studied.

Normally, bone mass increases, plateaus and then starts to decrease with aging, increasing the chances of bone fractures and osteoporosis. Osteogenesis has been reported to decrease at increasing ages in AT-MSCs [20, 33] and BM-MSCs [23]. Our data showed significant age-related differences in the osteogenic potential of PDMSCs, as measured by real-time RT-qPCR, using osteocalcin gene expression and Alizarin staining; group D showed the highest osteocalcin gene expression, followed by a significant drop in osteogenic potential in group E. Similar results were shown by Zhu et al., who reported a decline in osteogenic potential starting in the middle age group (40-49 years) for AT-MSCs isolated from female donors [34]. Interestingly, in our study, Group A showed high osteogenesis potential but not chondrogenesis or adipogenesis potential, which could be explained by the circulating estrogen levels in female donors, which have been shown to regulate the osteogenic potential of stem cells in females [35, 36]. Further studies are required to correlate estrogen levels and osteogenic differentiation potential at different maternal ages. Altogether, our osteogenic differentiation results combined with our chondrogenic potential findings indicate that PDMSCs from donors 31-35 years could be a better source for future orthopedic applications.

Telomere length is an important indicator of cellular senescence and replicative ability in cells. Studies have shown that in hMSCs, telomerase activity is undetectable, and telomere length gradually shortens with each cell replication [37]. Stem cells with multilineage differentiation potential and self-renewal capacity, such as PDMSCs, have mechanisms to maintain telomere length [38]. The use of adult-derived stem cells, such as AT-MSCs and BM-MSCs, is limited in clinical applications for cell therapy due to age-related cellular changes; hence, determining telomere length in PDMSCs may help characterize and understand biological differences. Here, we report that PDMSCs from mothers of median ages (22-35 years) exhibited shorter telomeres than PDMSCs from mothers aged 18-21 and >36 years, which exhibited longer telomeres. Our results revealed a negative correlation between telomere length and cell proliferation; groups A and E showed longer telomeres and a decreased cellular proliferation rate, and groups B, C and D presented shorter telomeres with an increased cellular proliferation rate, suggesting a correlation between cell proliferation and telomere shortening (Figure 4B). Our results are consistent with previous observations in fetal membrane-derived MSCs from sources other than the placenta from three donor age groups (GI: 20-29 years, GII: 30-39 years and GIII: 40 and over), in which decreased telomere length was reported in groups with increased proliferation rates [39].

In conclusion, this study provides a thorough characterization of the biological effect of maternal donor age on the regenerative potential of MSCs isolated from chorionic villi of placental tissue. The results demonstrated correlations of age and proliferation, self-renewal, and differentiation capacity and telomere length. Moreover, the age groups studied involved shorter age ranges (Group A: 18-21 years; Group B: 22-25 years; Group C: 26-30 years; Group D: 31-35 years; Group E: 36 years and older), which allowed us to obtain a better understanding regarding the maternal age range that presented higher regenerative potential. Altogether, the results from the present study suggest that PDMSCs from median maternal ages (22-35 years) present higher regenerative capacity in terms of self-renewal, proliferative, and differentiation capacity than do PDMSCs from younger (18-21 years) and older (36 years and older) donors. Interestingly, our results also showed that PDMSCs from donors of median maternal ages with higher proliferative rates present shorter telomeres than PDMSCs from younger (18-21 years) and older (36 years and older) donors, which have reduced self-renewal ability and longer telomeres. To our knowledge, this is the first report regarding the effect of maternal age on the differential expression of multipotency and pluripotency markers in *in vitro*-cultured PDMSCs and how it correlates with proliferation state and telomere length.

The translation of our study will provide valuable information regarding the use of autologous MSCs or adult-derived stem cells from maternal donors of targeted ages, which may potentiate its efficacy for future use in regenerative medicine research and clinical applications.

## MATERIALS AND METHODS

### PDMSC isolation, expansion, and maternal age grouping

Human placentas were obtained from full-term pregnancies (38~40 weeks of gestation) with no evidence of prior chronic disease or obstetrical and surgical complications. Informed consent forms were signed by all of the participants after an explanation of the study was provided. This study was approved by the National Committee on Research Bioethics and the Institutional Review Boards of Hospital Santo Tomas and Hospital Nacional. All procedures were in compliance with the Declaration of Helsinki.

MSCs were isolated from the chorionic villi of freshly delivered placentas, as previously published [40]. Briefly, full-term human placentas were obtained from mothers with informed consent, and cell isolation initiated within 40 minutes after placental collection. Chorionic villi tissues were harvested and minced for digestion with Collagenase Type 2 at 37° C for 40min. The digested tissues were filtered and used for density gradient centrifugation to isolate mononuclear cells. Flow cytometry results revealed specific staining for each of the positive markers of MSC stemness, while the expression of the negative markers CD45 and CD3 (Figure 2C) was undetectable, validating the quality of our isolated cells. Isolated cells were seeded at 1 × 10^4^ per cm^2^ in Dulbecco’s modified Eagle’s medium (DMEM)-low glucose with 10% fetal bovine serum (Gibco BRL, Life Technologies, NY, USA) at 37° C with 5% CO_2_. PDMSCs (P4, n=3) from three different donors were grouped for pooling according to their respective maternal age (Table 1). After the cells reached 80% confluence, the PDMSCs were seeded on a polystyrene surface for further culture [41, 42].

**Table 1:** Maternal age ranges used for PDMSCs grouping.

**Group**	**Age**
A	18-21
B	22-25
C	26-30
D	31-35
E	36 and over

### Proliferation assays, generation time studies, and cell growth curves

To determine the cumulative population doubling (CPD) of PDMSCs, cells were seeded at a cell density of 1 × 10^4^ per cm^2^ and subcultured approximately every 4 to 5 days. The CPD was plotted against time and calculated using the equation ΔPD=log (final total cell number/initial cell number seeded)/log2 [42].

A cell growth curve was generated by seeding the cells at a cell density of 1 × 10^4^ per cm^2^ and then plotting cell number against time. Cell counts were performed using a hematocytometer every 48 hours during a 10-day culture period [42].

PDMSCs derived from the 5 donors age groups were seeded in a 96-well ELISA plate in triplicates. After cells reached 80% confluence MTT assay was performed using Thiazolyl Blue Tetrazolium Bromide (#M5655, SIGMA) diluted in PBS and filtered before used. 50μl of MTT working solution was added to each well and incubated for 4 hours at 37° C with 5% CO2. After incubation, 100μl of detergent reagent was added. Plate was gently shaken to dissolve crystals and absorbance was measure at 570 nm in a microplate reader.

### Colony-forming unit-fibroblast (CFU-F) assay

Cells were seeded at a density of 1000 cells/per well in 12-well plates in DMEM supplemented with 10% FBS, and each group was seeded in triplicate. After 8 days of culture, the cells were washed with DPBS, fixed with formaldehyde at 4% and stained with crystal violet for 5 minutes followed by a ddH_2_O wash. Colonies with < 40 cells were counted using a hemocytometer [43].

### Placenta-derived mesenchymal stem cell marker analysis

The effect of maternal age on the expression of multipotency and pluripotency markers in PDMSCs was analyzed by flow cytometry. PDMSCs were washed with Dulbecco’s phosphate-buffered saline (DPBS, Gibco) and resuspended in fluorescence activated cell sorting (FACS) buffer (Thermo Fisher Scientific) with FITC-conjugated anti-human CD90, PE-conjugated anti-human CD73, and APC-conjugated anti-human CD105 (BioLegend, CA, USA) for 20 minutes at room temperature in the dark. The cells were then washed with FACS buffer and resuspended in fixation buffer (Thermo Fisher Scientific). For intracellular staining, the cell membrane was permeabilized with 1x Perm buffer (BioLegend) for 20 minutes at room temperature. The cells were resuspended in 1x Perm buffer with PE-conjugated anti-human OCT4, FITC-conjugated anti-human SSEA4 and Alexa Fluor® 647-conjugated anti-NANOG antibodies for 2 hours in the dark. The cells were then washed and resuspended in fixation buffer. The fluorescence intensity of 20,000 cells was recorded on a flow cytometer (Guava, Merck) and analyzed using FlowJo software with excitation/emission wavelengths 494/520 for FITC, 496/578 for PE, 650/660 for APC and 650/660 for Alexa Fluor® 647.

### Reverse transcription-quantitative polymerase chain reaction

Total RNA was extracted from cell cultures according to the guidelines of the RNeasy Mini Kit manual (Qiagen). Two micrograms of RNA were used to generate cDNA by reverse transcription with SuperScript™ III Reverse Transcriptase according to the manufacturer's instructions. Quantitative real-time polymerase chain reaction (RT-qPCR) was performed using SYBR® Green RT-qPCR master mix (Applied BioSystems, MA, USA). Gene expression levels in the cells were normalized to the housekeeping gene glyceraldehyde 3-phosphate dehydrogenase (GAPDH). The results are expressed as fold changes relative to the controls. Primer details are listed in Table 2.

**Table 2:** Oligonucleotide sequence of primers.

**Gene**	**Sequence**
OCT4	AGGGCAAGCGATCAAGCA
	GGAAAGGGACCGAGGAGTA
NANOG	TAGCAATGGTGTGACGCAGG
	TGTCTGTGACTGGAGTTGTGT
Sox2	GGACAGTTACGCGCACATGA
	AGCCGTTCATGTAGGTCTGC
ACAN	CAGTCGAAACAGCCACCTCC
	TCTGTCTCCTTGCAGGTCCC
SOX9	GCTCTGGAGACTTCTGAACGAGAG
	CGTTCTTCACCGACTTCCTCC
ALBP	TAGATGGGGGTGTCCTGGTA
	GCTAGAAGATACTCACCACCAC
FABP4	ACGAGAGGATGATAAACTGGTGG
	GCGAACTTCAGTCCAGGTCAAC
CEBPB	AGAAGACCGTGGACAAGCACAG
	CTCCAGGACCTTGTGCTGCGT
Osteocalcin	CTTTGGGGTTTGGCCTACGG
	CCTTTTCTCTCACCCCAGCCATT
COL1	CGTGGCAGTGATGGAAGTG
	AGCAGGACCAGCGTTACC
RUNX2	GGAATGCCTCTGCTGTTATG
	TTCTGTCTGTGCCTTCTGG
OPN	CGAGGTGATAGTGTGGTTTATGG
	GCACCATTCAACTCCTCGCTTTC
GAPDH	AGCCACATCGCTCAGACACC
	GTACTCAGCGGCCAGCATCG

### *In vitro* induction of differentiation

Adipogenic, osteogenic and chondrogenic induction of PDMSCs was performed according to manufacturer’s instructions in the StemPro® Adipogenesis, Osteogenesis, and Chondrogenesis Differentiation Kit (Gibco). PDMSCs at passage 2 were seeded on polystyrene surfaces (control) and cultured in low-glucose DMEM-10% FBS for 3 days prior to induction of differentiation. For adipogenic induction, PDMSCs were cultured on StemPro® Adipocyte Differentiation Basal Medium supplemented with StemPro® adipogenesis supplement and gentamycin (10 mg/mL, Gibco) for 7 days. Adipogenesis was assessed by Oil Red O staining (Sigma-Aldrich, MO, USA). For induction of chondrogenic differentiation, PDMSCs were cultured in StemPro® Chondrocyte Differentiation Basal Medium with StemPro® chondrogenesis supplement and gentamycin reagent (10 mg/mL) for 12 days. Chondrogenesis was assessed with 1% Alcian blue stain (Sigma-Aldrich) prepared in 0.1 N HCL. For induction of osteogenic differentiation, PDMSCs were cultured in StemPro® Osteocyte Differentiation Basal Medium with StemPro® osteogenesis supplement and gentamycin reagent (10 mg/mL) for 13 days. Osteogenesis was assessed by Alizarin Red S staining (Sigma-Aldrich). Cells were fixed prior to staining. Images were captured with an inverted light microscope (Leica Microsystems, IL, USA).

### Genomic DNA extraction and evaluation of telomere length by RT-qPCR

Genomic DNA was isolated using the Qiagen DNeasy Blood and Tissue Kit (Qiagen) according to the manufacturer’s instructions. Telomere length was evaluated by adding 5 ng of DNA into the Absolute Human Telomere Length Quantification PCR Assay Kit (ScienceCells, CA, USA) with SYBR® Green RT-qPCR master mix (Applied BioSystems) according to the manufacturer’s protocol. The 2^-∆∆Cq^ method was used to calculate the average telomere length. The change in telomere length was derived from the reference sample telomere length (695 ± 16 kb) x 2^-∆∆Cq^.

### Statistical analysis

All experiments were performed at least in triplicate (n=3). Experimental results are expressed as the mean SD of the samples. Statistical analysis was performed using one-way analysis of variance with Tukey’s multiple comparison test. Homogeneity of variance was assumed with a 95% confidence interval level. The results for comparisons with at least n=3 and *p*<0.05 were considered significant. All statistical analyses were performed using GraphPad Prism software Version 6.0.

## Supplementary Material

Supplementary Figures

## References

[1] Liras A. Future research and therapeutic applications of human stem cells: general, regulatory, and bioethical aspects. J Transl Med. 2010; 8:131. 10.1186/1479-5876-8-131 21143967 PMC3014893

[2] Lv FJ, Tuan RS, Cheung KM, Leung VY. Concise review: the surface markers and identity of human mesenchymal stem cells. Stem Cells. 2014; 32:1408–19. 10.1002/stem.1681 24578244

[3] Stanko P, Kaiserova K, Altanerova V, Altaner C. Comparison of human mesenchymal stem cells derived from dental pulp, bone marrow, adipose tissue, and umbilical cord tissue by gene expression. Biomed Pap Med Fac Univ Palacky Olomouc Czech Repub. 2014; 158:373–77. 10.5507/bp.2013.078 24145770

[4] Antoniadou E, David AL. Placental stem cells. Best Pract Res Clin Obstet Gynaecol. 2016; 31:13–29. 10.1016/j.bpobgyn.2015.08.014 26547389

[5] Marcus AJ, Woodbury D. Fetal stem cells from extra-embryonic tissues: do not discard. J Cell Mol Med. 2008; 12:730–42. 10.1111/j.1582-4934.2008.00221.x 18194447 PMC4401124

[6] Fierabracci A, Lazzari L, Muraca M, Parolini O. How far are we from the clinical use of placental-derived mesenchymal stem cells? Expert Opin Biol Ther. 2015; 15:613–17. 10.1517/14712598.2015.1000856 25556657

[7] Janzen V, Forkert R, Fleming HE, Saito Y, Waring MT, Dombkowski DM, Cheng T, DePinho RA, Sharpless NE, Scadden DT. Stem-cell ageing modified by the cyclin-dependent kinase inhibitor p16INK4a. Nature. 2006; 443:421–26. 10.1038/nature05159 16957735

[8] Jeon YJ, Kim J, Cho JH, Chung HM, Chae JI. Comparative Analysis of Human Mesenchymal Stem Cells Derived From Bone Marrow, Placenta, and Adipose Tissue as Sources of Cell Therapy. J Cell Biochem. 2016; 117:1112–25. 10.1002/jcb.25395 26448537

[9] Iezzi I, Cerqueni G, Licini C, Lucarini G, Mattioli Belmonte M. Dental pulp stem cells senescence and regenerative potential relationship. J Cell Physiol. 2019; 234:7186–97. 10.1002/jcp.27472 30362542

[10] Alrefaei GI, Al-Karim S, Ayuob NN, Ali SS. Does the maternal age affect the mesenchymal stem cell markers and gene expression in the human placenta? What is the evidence? Tissue Cell. 2015; 47:406–19. 10.1016/j.tice.2015.05.005 26067657

[11] Zhao P, Ise H, Hongo M, Ota M, Konishi I, Nikaido T. Human amniotic mesenchymal cells have some characteristics of cardiomyocytes. Transplantation. 2005; 79:528–35. 10.1097/01.tp.0000149503.92433.39 15753841

[12] Parolini O, Alviano F, Bagnara GP, Bilic G, Bühring HJ, Evangelista M, Hennerbichler S, Liu B, Magatti M, Mao N, Miki T, Marongiu F, Nakajima H, et al. Concise review: isolation and characterization of cells from human term placenta: outcome of the first international Workshop on Placenta Derived Stem Cells. Stem Cells. 2008; 26:300–11. 10.1634/stemcells.2007-0594 17975221

[13] Fan M, Chen W, Liu W, Du GQ, Jiang SL, Tian WC, Sun L, Li RK, Tian H. The effect of age on the efficacy of human mesenchymal stem cell transplantation after a myocardial infarction. Rejuvenation Res. 2010; 13:429–38. 10.1089/rej.2009.0986 20583954

[14] Pasquinelli G, Tazzari P, Ricci F, Vaselli C, Buzzi M, Conte R, Orrico C, Foroni L, Stella A, Alviano F, Bagnara GP, Lucarelli E. Ultrastructural characteristics of human mesenchymal stromal (stem) cells derived from bone marrow and term placenta. Ultrastruct Pathol. 2007; 31:23–31. 10.1080/01913120601169477 17455095

[15] Alviano F, Fossati V, Marchionni C, Arpinati M, Bonsi L, Franchina M, Lanzoni G, Cantoni S, Cavallini C, Bianchi F, Tazzari PL, Pasquinelli G, Foroni L, et al. Term Amniotic membrane is a high throughput source for multipotent Mesenchymal Stem Cells with the ability to differentiate into endothelial cells *in vitro*. BMC Dev Biol. 2007; 7:11. 10.1186/1471-213X-7-11 17313666 PMC1810523

[16] Bhandi S, Alkahtani A, Reda R, Mashyakhy M, Boreak N, Maganur PC, Vishwanathaiah S, Mehta D, Vyas N, Patil V, Raj AT, Testarelli L, Patil S. Parathyroid Hormone Secretion and Receptor Expression Determine the Age-Related Degree of Osteogenic Differentiation in Dental Pulp Stem Cells. J Pers Med. 2021; 11:349. 10.3390/jpm11050349 33925324 PMC8144966

[17] Soncini M, Vertua E, Gibelli L, Zorzi F, Denegri M, Albertini A, Wengler GS, Parolini O. Isolation and characterization of mesenchymal cells from human fetal membranes. J Tissue Eng Regen Med. 2007; 1:296–305. 10.1002/term.40 18038420

[18] Sperka T, Wang J, Rudolph KL. DNA damage checkpoints in stem cells, ageing and cancer. Nat Rev Mol Cell Biol. 2012; 13:579–90. 10.1038/nrm3420 22914294

[19] Müezzinler A, Zaineddin AK, Brenner H. A systematic review of leukocyte telomere length and age in adults. Ageing Res Rev. 2013; 12:509–19. 10.1016/j.arr.2013.01.003 23333817

[20] Choudhery MS, Badowski M, Muise A, Pierce J, Harris DT. Donor age negatively impacts adipose tissue-derived mesenchymal stem cell expansion and differentiation. J Transl Med. 2014; 12:8. 10.1186/1479-5876-12-8 24397850 PMC3895760

[21] Varghese J, Griffin M, Mosahebi A, Butler P. Systematic review of patient factors affecting adipose stem cell viability and function: implications for regenerative therapy. Stem Cell Res Ther. 2017; 8:45. 10.1186/s13287-017-0483-8 28241882 PMC5329955

[22] Naz S, Khan FR, Zohra RR, Lakhundi SS, Khan MS, Mohammed N, Ahmad T. Isolation and culture of dental pulp stem cells from permanent and deciduous teeth. Pak J Med Sci. 2019; 35:997–1002. 10.12669/pjms.35.4.540 31372131 PMC6659089

[23] Zaim M, Karaman S, Cetin G, Isik S. Donor age and long-term culture affect differentiation and proliferation of human bone marrow mesenchymal stem cells. Ann Hematol. 2012; 91:1175–86. 10.1007/s00277-012-1438-x 22395436

[24] Borzou B, Mehrabani D, Zare S, Zamani-Pereshkaft M, Acker JP. The Effect of Age and Type of Media on Growth Kinetics of Human Amniotic Fluid Stem Cells. Biopreserv Biobank. 2020; 18:389–94. 10.1089/bio.2019.0103 32799559

[25] Abumaree MH, Al Jumah MA, Kalionis B, Jawdat D, Al Khaldi A, AlTalabani AA, Knawy BA. Phenotypic and functional characterization of mesenchymal stem cells from chorionic villi of human term placenta. Stem Cell Rev Rep. 2013; 9:16–31. 10.1007/s12015-012-9385-4 22628114

[26] Alrefaei GI, Ayuob NN, Ali SS, Al-Karim S. Effects of maternal age on the expression of mesenchymal stem cell markers in the components of human umbilical cord. Folia Histochem Cytobiol. 2015; 53:259–71. 10.5603/FHC.a2015.0022 26339985

[27] Musiał-Wysocka A, Kot M, Sułkowski M, Badyra B, Majka M. Molecular and Functional Verification of Wharton’s Jelly Mesenchymal Stem Cells (WJ-MSCs) Pluripotency. Int J Mol Sci. 2019; 20:1807. 10.3390/ijms20081807 31013696 PMC6515095

[28] Tantrawatpan C, Manochantr S, Kheolamai P, U-Pratya Y, Supokawej A, Issaragrisil S. Pluripotent gene expression in mesenchymal stem cells from human umbilical cord Wharton’s jelly and their differentiation potential to neural-like cells. J Med Assoc Thai. 2013; 96:1208–17. 24163998

[29] Gao LR, Zhang NK, Ding QA, Chen HY, Hu X, Jiang S, Li TC, Chen Y, Wang ZG, Ye Y, Zhu ZM. Common expression of stemness molecular markers and early cardiac transcription factors in human Wharton’s jelly-derived mesenchymal stem cells and embryonic stem cells. Cell Transplant. 2013; 22:1883–900. 10.3727/096368912X662444 23394400

[30] Fong CY, Chak LL, Biswas A, Tan JH, Gauthaman K, Chan WK, Bongso A. Human Wharton’s jelly stem cells have unique transcriptome profiles compared to human embryonic stem cells and other mesenchymal stem cells. Stem Cell Rev Rep. 2011; 7:1–16. 10.1007/s12015-010-9166-x 20602182

[31] Trivanović D, Jauković A, Popović B, Krstić J, Mojsilović S, Okić-Djordjević I, Kukolj T, Obradović H, Santibanez JF, Bugarski D. Mesenchymal stem cells of different origin: Comparative evaluation of proliferative capacity, telomere length and pluripotency marker expression. Life Sci. 2015; 141:61–73. 10.1016/j.lfs.2015.09.019 26408916

[32] Wagner W, Wein F, Seckinger A, Frankhauser M, Wirkner U, Krause U, Blake J, Schwager C, Eckstein V, Ansorge W, Ho AD. Comparative characteristics of mesenchymal stem cells from human bone marrow, adipose tissue, and umbilical cord blood. Exp Hematol. 2005; 33:1402–16. 10.1016/j.exphem.2005.07.003 16263424

[33] Liu M, Lei H, Dong P, Fu X, Yang Z, Yang Y, Ma J, Liu X, Cao Y, Xiao R. Adipose-Derived Mesenchymal Stem Cells from the Elderly Exhibit Decreased Migration and Differentiation Abilities with Senescent Properties. Cell Transplant. 2017; 26:1505–19. 10.1177/0963689717721221 29113467 PMC5680952

[34] Zhu M, Kohan E, Bradley J, Hedrick M, Benhaim P, Zuk P. The effect of age on osteogenic, adipogenic and proliferative potential of female adipose-derived stem cells. J Tissue Eng Regen Med. 2009; 3:290–301. 10.1002/term.165 19309766

[35] Robinson JA, Harris SA, Riggs BL, Spelsberg TC. Estrogen regulation of human osteoblastic cell proliferation and differentiation. Endocrinology. 1997; 138:2919–27. 10.1210/endo.138.7.5277 9202236

[36] Ankrom MA, Patterson JA, d’Avis PY, Vetter UK, Blackman MR, Sponseller PD, Tayback M, Robey PG, Shapiro JR, Fedarko NS. Age-related changes in human oestrogen receptor alpha function and levels in osteoblasts. Biochem J. 1998; 333:787–94. 10.1042/bj3330787 9677341 PMC1219645

[37] Fehrer C, Lepperdinger G. Mesenchymal stem cell aging. Exp Gerontol. 2005; 40:926–30. 10.1016/j.exger.2005.07.006 16125890

[38] Fathi E, Charoudeh HN, Sanaat Z, Farahzadi R. Telomere shortening as a hallmark of stem cell senescence. Stem Cell Investig. 2019; 6:7. 10.21037/sci.2019.02.04 31019963 PMC6458335

[39] Correction to: Impact of Mothers’ Age on Telomere Length and Human Telomerase Reverse Transcriptase Expression in Human Fetal Membrane-Derived Mesenchymal Stem Cells by Alrefaei GI, Alkarim SA, and Abduljabbar HS. Stem Cells Dev 2019;28;24;1632-1645 DOI:10.1089/scd.2019-0144. Stem Cells Dev. 2020; 29:380–81. 10.1089/scd.2019.0144.correx 31650883

[40] Liu CM, Chang CH, Yu CH, Hsu CC, Huang LL. Hyaluronan substratum induces multidrug resistance in human mesenchymal stem cells via CD44 signaling. Cell Tissue Res. 2009; 336:465–75. 10.1007/s00441-009-0780-3 19350274

[41] Widholz B, Tsitlakidis S, Reible B, Moghaddam A, Westhauser F. Pooling of Patient-Derived Mesenchymal Stromal Cells Reduces Inter-Individual Confounder-Associated Variation without Negative Impact on Cell Viability, Proliferation and Osteogenic Differentiation. Cells. 2019; 8:633. 10.3390/cells8060633 31238494 PMC6628337

[42] Wong TY, Chen YH, Liu SH, Solis MA, Yu CH, Chang CH, Huang LL. Differential Proteomic Analysis of Human Placenta-Derived Mesenchymal Stem Cells Cultured on Normal Tissue Culture Surface and Hyaluronan-Coated Surface. Stem Cells Int. 2016; 2016:2809192. 10.1155/2016/2809192 27057169 PMC4709773

[43] Huang S, Feng C, Wu Y, Yang S, Ma K, Wu X, Fu X. Dissimilar characteristics of umbilical cord mesenchymal stem cells from donors of different ages. Cell Tissue Bank. 2013; 14:707–13. 10.1007/s10561-013-9364-2 23475054

