# Supplementary material for: Increased proliferation and differentiation capacity of placenta-derived mesenchymal stem cells from women of median maternal age correlates with telomere shortening
Source: Aging (Albany NY). 2021 Nov 29;13(22):24542–59. doi: 10.18632/aging.203724 (PMC8660609; doi:10.18632/aging.203724)
Supplement: Supplementary Figures [file aging-13-203724-s001.pdf]

SUPPLEMENTARY FIGURES

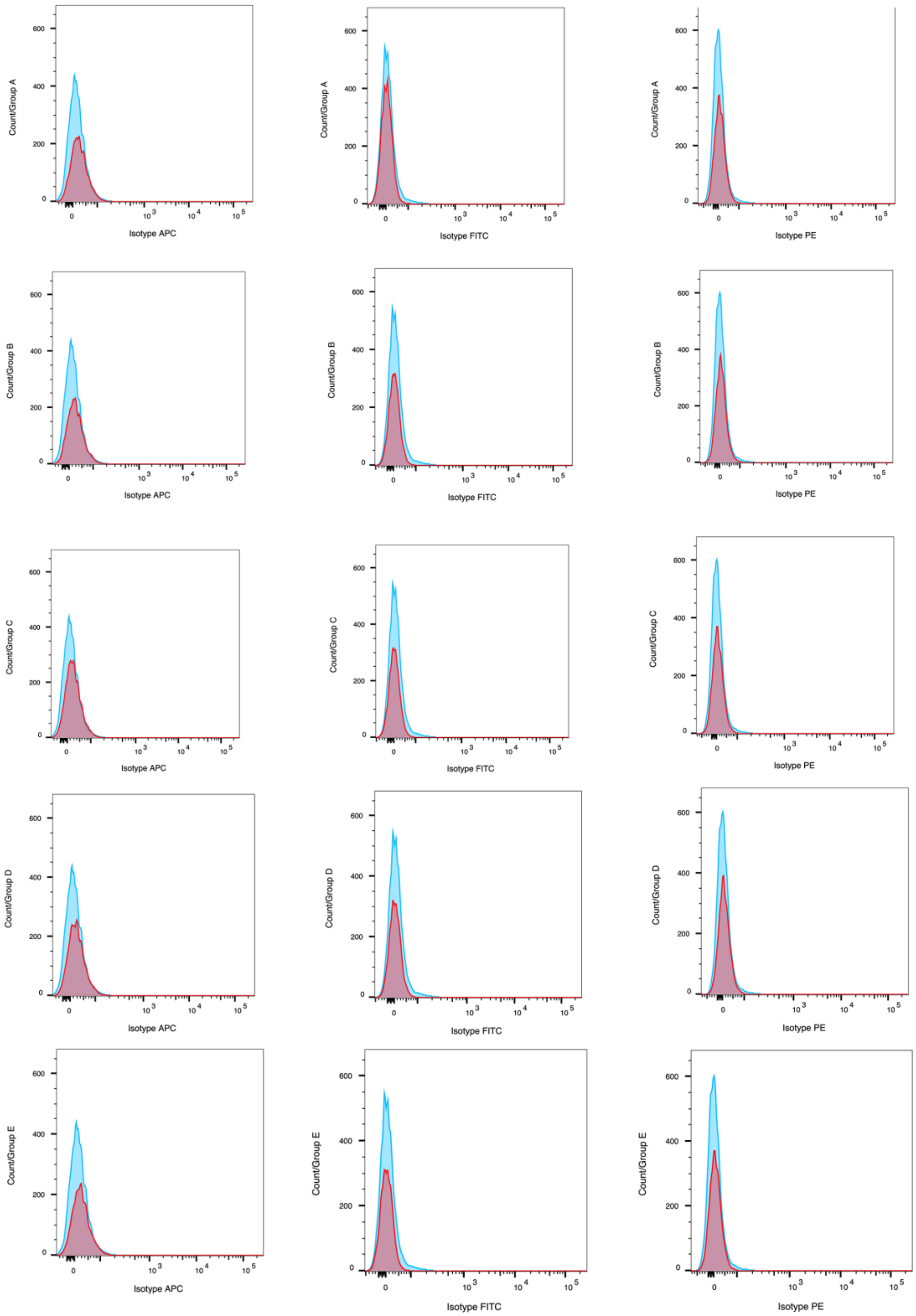

Supplementary Figure 1. Representative histograms by FACS demonstrates no expression of isotype controls for APC, PE, and FITC in any of the 5 maternal age groups. Group A: 18-21; Group B: 22-25; Group C: 26-30; Group D: 31-35; Group E: 36 and over.

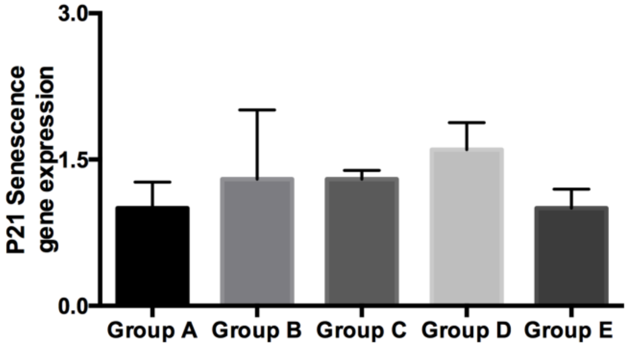

Supplementary Figure 2. Graphs showing no difference in the gene expression of the senescence marker, p21, by real time PCR of all 5 age groups. Group A: 18-21; Group B: 22-25; Group C: 26-30; Group D: 31-35; Group E: 36 and over.
